# *Salmonella* Effector SpvB Inhibits NF-κB Activity *via* KEAP1-Mediated Downregulation of IKKβ

**DOI:** 10.3389/fcimb.2021.641412

**Published:** 2021-03-18

**Authors:** Sidi Yang, Qifeng Deng, Lanqing Sun, Yuan Zhu, Kedi Dong, Shuyan Wu, Rui Huang, Yuanyuan Li

**Affiliations:** ^1^ Department of Medical Microbiology, Medical College of Soochow University, Suzhou, China; ^2^ School of Medicine, Sun Yat-sen University, Guangzhou, China

**Keywords:** *Salmonella*, SpvB, NF-κB, IKKβ, KEAP1

## Abstract

Bacterial pathogens have a broad arsenal of genes that are tightly regulated and coordinated to facilitate adaptation to alter host inflammatory response and prolong intracellular bacterial survival. *Salmonella enterica* serovar Typhimurium utilizes a type III secretion system (T3SS) to deliver effector molecules into host cells and regulate signal transduction pathways such as NF-κB, thereby resulting in salmonellosis. SpvB, a pSLT-encoded cytotoxic protein secreted by *Salmonella* pathogenicity island-2 T3SS, is associated with enhanced *Salmonella* survival and intracellular replication. In this report, we characterized the effects of SpvB on NF-κB signaling pathway. We showed that SpvB has a potent and specific ability to prevent NF-κB activation by targeting IκB kinase β (IKKβ). Previous studies from our laboratory showed that SpvB decreases Nrf2 through its C-terminal domain. Here we further demonstrated that KEAP1, a cytoplasmic protein that interacts with Nrf2 and mediates its proteasomal degradation, is involved in SpvB-induced downregulation of IKKβ expression and phosphorylation. Reduction of KEAP1 by small-interfering RNA prevented the suppression of IKKβ and its phosphorylation mediated by SpvB. These findings revealed a novel mechanism by which *Salmonella* modulates NF-κB activity to ultimately facilitate intracellular bacterial survival and proliferation and delay host immune response to establish infection.

## Introduction

Early sensing of pathogenic bacteria by the host numerous innate and adaptive immune mechanisms is important to limit microbial intrusion. By eliciting inflammation, pathogenic bacteria use highly evolved mechanisms to counteract host defense mechanisms (Baumler and Sperandio, 2016). During the long-standing associations with the host, pathogens have developed complex adaptations to modulate host inflammatory response to promote their own growth and virulence. A facultative intracellular pathogen *Salmonella enterica* serovar Typhimurium (*S.* Typhimurium), which is a leading cause of food- and water-borne diseases in mammals, has acquired adaptations that interfere with host defense mechanisms (Besser, 2018). One important adaptation of *Salmonella* is the type III secretion system (T3SS), which delivers effector molecules into host cells. There are two T3SSs encoded by *Salmonella* pathogenicity islands-1 and -2 (SPI-1 and SPI-2), which deliver more than 40 effectors into target cells to facilitate bacterial invasion, survival, and proliferation (Agbor and Mccormick, 2011). Several effector proteins have been shown to interfere with the activity of host inflammatory signaling pathways, including nuclear factor κB (NF-κB) and mitogen-activated protein kinases (MAPK), which result in attenuated host inflammatory response and enhanced bacterial replication within host cells (Kurtz et al., 2017).

NF-κB signaling pathway is a major innate immune signaling pathway responsible for early detection of pathogens such as *Salmonella* (Pinaud et al., 2018). Under naive conditions, NF-κB remains inactive in the cytoplasm bound to the NF-κB inhibitor alpha (IκBα) protein. Following stimulation with multiple exogenous and endogenous substances, which are sensed by tumor necrosis factor receptor (TNFR), interleukin-1 receptor (IL-1R), toll-like receptors (TLRs), and NOD-like receptors (NLRs) (Verstrepen et al., 2008; Caruso et al., 2014; Keestra-Gounder et al., 2015), different pathways are triggered to lead to phosphorylation of the inhibitor of nuclear factor-κB kinases (IKKs). The IKK complex contains three subunits including IKKα, IKKβ, and a regulatory protein, NEMO (or IKKγ). In particular, IKKβ activation is dependent on the phosphorylation of the kinase domain. IKKβ activation can further phosphorylate IκBα and lead to its degradation, thereby releasing p50/p65 for import into nucleus to initiate transcription of the target genes (Bhoj and Chen, 2009). Numerous components of the NF-κB signaling pathway are targeted by some of the *Salmonella* effectors (such as SseK1, SspH1, GtgA, and AvrA) that result in inhibition of the NF-kB pathway (Collier-Hyams et al., 2002; Haraga and Miller, 2003; Sun et al., 2016; Gunster et al., 2017). Although several effectors delivered by *S*. Typhimurium T3SS can efficiently attenuate NF-kB activation, inhibit the host inflammatory response, and promote bacterial replication in host cells and tissues, the functions and the underlying mechanisms of most effectors remain obscure.

*Salmonella* plasmid virulence (*spv*) gene, a highly conserved 8-kb region in the pSLT virulence plasmid from non-typhoid *Salmonella* strains of clinically significant serovars, has been implicated in intracellular survival and growth (Fabrega and Vila, 2013). The *spv* region contains a positive transcriptional regulator gene *spvR*, a negative regulator gene *spvA*, and three structural genes *spvB*, *spvC*, and *spvD* (Guiney and Fierer, 2011; Passaris et al., 2018). Previous studies have found that all the *spv* effectors are secreted *via* the T3SS, and *spv*-containing *Salmonella* exhibit enhanced intracellular proliferation in intestinal and extraintestinal tissues of the natural host (Libby et al., 1997), suggesting that they have the potential to target molecules and mechanisms to attenuate host inflammatory response to establish a successful infection. Each of the *spv* effectors has been shown to target specific signal transduction pathways in a variety of cell types (Passaris et al., 2018).

Here, we found that *spv* could inhibit NF-κB activation during *Salmonella* infection, and SpvB, a member of the *spv* effectors, is critical for *spv*-mediated NF-κB inhibition and IκBα degradation. The detailed investigation of the NF-kB signaling pathway suggested that SpvB acts at the level of IKKβ activation. We showed that SpvB prevents the expression and phosphorylation of IKKβ, and this was associated with SpvB-mediated upregulation of an E3 ligase KEAP1. Together, these findings indicated that *Salmonella* effector protein SpvB inhibits the activation of NF-κB signaling pathway *via* repressing the activity of IKKβ by targeting KEAP1. Our studies revealed a novel inhibitory mechanism used by *Salmonella* to disrupt host inflammatory response to enhance its proliferation and virulence.

## Materials and Methods

### Bacterial Strains and Growth Condition

Wild-type (WT) *S*. Typhimurium strain SL1344 was kindly provided by Professor Qian Yang (Nanjing Agricultural University, Nanjing, China). The *ΔspvB* strain was constructed in our previous work (Yang et al., 2019). *Δspv*, *ΔspvA*, *ΔspvD* and *ΔspvBD* mutant strains were constructed with the λ-Red-mediated recombination system as previously described (Datsenko and Wanner, 2000). *ΔspvBD*,p*spvB* and *ΔspvBD*,p*spvD* strains were complemented with *ΔspvBD* in plasmid pBAD/gIIIA (Invitrogen, Carlsbad, CA, USA) encoding the full-length of *spvB* or *spvD* gene. The primers used in this study are listed in **Table 1**. Bacteria were grown in Luria-Bertani (LB) medium (Hangwei, Hangzhou, China) at 37°C and supplemented with ampicillin (100 μg/ml) or kanamycin (50 μg/ml) as appropriate.

**Table 1:** Primers used for the bacterial strain construction.

Primer	Sequence, 5’-3’	
	Forward	Reverse
*Δspv*-kan	AGTCAGGGTATGCATAAAATCTATCGCCCATAATCCTATCCAGTAACCCCGTGTAGGCTGGAGCTGCTTC	GACAGAATACGCCGGAGTAGCCTGCCCTAAAGCCCTTCGGGCTTTTCGCCATGGGAATTAGCCATGGTCC
*ΔspvA*-kan	ATCAGTCTTCAGGATTTCATTCTGTTTATTTTCAGGAGTCATCATTATTTGTGTAGGCTGGAGCTGCTTC	TCAGATTCTGCAGAATGGTCTGCTGCTCACTACTCTGGTAGCGCGGGAAGATGGGAATTAGCCATGGTCC
*ΔspvB*-kan	CAGAAAATATACCTGGCCATCGTCAGACGGCCAGTTTCAGGAGATAGTGTGTGTAGGCTGGAGCTGCTTC	CCTTATAGAGCTAGGCCGCTCATACCACTTCTGGAATAGATTCTTAGTATATGGGAATTAGCCATGGTCC
*ΔspvD*-kan	ATATTATATTTAATGTTTTGTAATATGCATTTTATTGAGGTAGTGTAACTGTGTAGGCTGGAGCTGCTTC	AAGGCTCTCTATTAACTTACCATTCATAAAATGAATATTTAAAAAAGTTAATGGGAATTAGCCATGGTCC
*ΔspvBD*,p*spvB*	CCTCGAGTTGATACTAAATGGTTTTTC	GGAATTCCTGAGTTGAGTACCCTCATGT
*ΔspvBD*,p*spvD*	CGAGCTCAGAGTTTCTGGTAGTGCGTC	GGGTACCCATCGTGTTTTTCATCATAAG

### Cell Culture

HeLa (human epithelial cell line) cells and 293T (human embryonic kidney cell line) cells were purchased from the ATCC. 293T-TLR4 cells were a gift from Professor Jianfeng Dai (Soochow University, Suzhou, China). All these cells were maintained in complete Dulbecco’s modified Eagle’s medium (DMEM) (HyClone, Logan, Utah, USA) supplemented with 10% heat-inactivated fetal bovine serum (FBS) (Biological Industries, Kibbutz Beit-Haemek, Israel) and 1% penicillin-streptomycin (Beyotime, Shanghai, China) at 37°C in a humidified atmosphere of 5% CO_2_.

For siRNA knockdown of KEAP1, cells were transfected with KEAP1 siRNA or negative control siRNA (NC siRNA) using Lipofectamine RNAiMAX reagent (Thermo Fisher Scientific, Waltham, MA, USA) for 24 h according to the manufacturer’s instructions. Knockdown efficiency was assessed by Western blot with KEAP1 antibody.

### Bacterial Infection of Cells

Overnight *S*. Typhimurium cultures were diluted 1:100 in fresh LB medium and subcultured for an extra 3 h. The bacteria were then washed three times with PBS and quantified spectrophotometrically by determining the optical density at 600 nm along with viable plate counts. HeLa cells were seeded in complete DMEM without antibiotics 24 h prior to use. Cells were infected with the different *S*. Typhimurium strains at the multiplicity of infection (MOI) described in the figure legends. One hour later, cells were washed in PBS and treated with DMEM containing 10% heat-inactivated FBS and amikacin (100 μg/ml) (Sigma, Saint Louis, Missouri, USA) to kill extracellular bacteria. 2 h later, cells were washed and maintained in DMEM containing 10% heat-inactivated FBS and low concentration of amikacin (10 μg/ml) (Sigma).

### Immunoblotting

Cells were lysed in RIPA Buffer (Beyotime) containing a protease and phosphatase inhibitor mini tablet (Thermo Fisher Scientific, Waltham, MA, USA). After normalization of protein concentrations with a BCA assay kit (Beyotime), samples were separated by SDS PAGE and transferred onto polyvinylidene fluoride membranes (Millipore, Saint Louis, Missouri, USA). Membranes were blocked in 5% nonfat milk in TBST and probed with primary antibodies against p-P65 (Abcam, Cambridge, MA, USA), P65 (ImmunoWay, Plano, TX, USA), IKBα (Santa Cruz Biotechnology, Dallas, TX, USA), IKKβ (Proteintech, Rosemont, IL, USA), p-IKKβ (Cell Signaling Technology, Danvers, MA, USA), KEAP1 (Proteintech), FLAG (ImmunoWay), EGFP (Bioss, Beijing, China), HA (Proteintech), α-tubulin (Proteintech), β-ACTIN (Bioss), and GAPDH (Bioss). Primary antibodies were incubated overnight at 4°C, washed, and incubated for 1 h with HRP-coupled secondary antibodies at room temperature. The blots were then visualized with an enhanced chemiluminescence luminescence reagent (Meilunbio, Dalian, China). The amount of correlated proteins was analyzed by Image J software program.

### Immunofluorescence Microscopy

Following infection, cells seeded on glass coverslips were washed three times with PBS prior to be fixed in 4% paraformaldehyde for 20 min and permeabilized in 0.3% Triton X-100 for 10 min. After being blocked with 3% BSA, the coverslips were incubated with primary antibodies at 4°C overnight, developed with the appropriate Alexa Fluor^®^ 555 secondary antibody (Thermo Fisher Scientific) for 1 h and then stained with Hoechst 33258 (Beyotime) for 10 min. All photomicrographs were taken with a Nikon Eclipse Ni-U fluorescence microscope (Nikon Corporation, Tokyo, Japan) with NIS-Elements F (Nikon Corporation).

### Luciferase Reporter Assays

HeLa cells were seeded at a density of 5 x 10^4^ cells per well in 24-well plates for 16 h. Cells were transfected with pNFκB-Luc (Firefly luciferase, experimental reporter) and phRL-TK reporter (Renilla luciferase, internal control) plasmids (Clontech, USA) using Lipofectamine 2000 (Thermo Fisher Scientific). After 24 h, the cells were infected with different *Salmonella* strains and NF-κB activity was measured using the Dual Luciferase assay kit (Promega, Madison, WI, USA) according to the manufacturer’s instructions.

293T cells or 293T-TLR4 cells were seeded into 24-well plates. 24 h later, cells were transfected with SpvB-EGFP, phRL-TK, and pNF-κB-luc, pIL-8-luc or pIKKβ-luc. The IL-8 reporter plasmid or the IKKβ reporter plasmid was constructed by amplifying the human IL-8 promoter sequence (F: 5’-GGAGGTACCTACTTGCCCAGAAGCGAACA-3’; R: 5’-TTACTCGAGGGCCAGCTTGGAAGTCATGT-3’) or the IKKβ reporter sequence (F: 5’-GGAGGTACCCCTCACAACAACTCTGGGGT-3’; R: 5’-TTACTCGAGGGGAATCTTAACGCGGGCAG-3’) from the genomic DNA of HeLa cells, which was then inserted into the pGL3-basic reporter plasmid (Clontech). Equal amounts of empty vector were used as control for the transfection process. After 24 h, cells were treated with PMA (Sigma, 25 nM), TNF-α (Peprotech, 10 ng/ml), LPS (InvivoGen, 100 ng/ml) or IL-1β (Peprotech, 10 ng/ml) for 3 h. NF-κB activity or IL-8 activity was determined using the Dual Luciferase assay kit (Promega). SpvB-EGFP, phRL-TK and pNF-κB-luc were co-transfected into 293T cells, together with pCMV-HA-MyD88, pcDNA3.1-FLAG-TRAF6, pcDNA3.1-FLAG-TAK1, pCMV-TAB1-HA, pcDNA3.1-IKKα-HA, pcDNA3.1-IKKβ-FLAG or pcDNA3.1-FLAG-P65 plasmids (Public Protein/Plasmid Library). After 24 h post-transfection, cell extracts were prepared and the activation of NF-κB induced by overexpression of distinct signal molecule was detected by luciferase assay.

### Immunoprecipitation

293T cells were seeded in 6-well plates for 16 h. Cells were transfected with indicated DNA with Lipofectamine 2000. Three wells were used per condition. Cells were lysed in RIPA Buffer (Beyotime) containing a protease and phosphatase inhibitor mini tablet (Thermo Fisher Scientific). Lysates were centrifuged at 16000 g and the supernatants were harvested and incubated with protein A/G Plus-agarose (Santa Cruz Biotechnology) by rotation for 2 h at 4°C. The supernatants were then incubated with anti-HA or anti-FLAG antibody overnight at 4°C for immunoprecipitation. Immunoprecipitated complexes were then washed three times, probed to anti-FLAG, anti-EGFP or anti-HA and subjected to immunoblotting.

### Quantitative Real-Time PCR (RT-qPCR)

Total RNA was isolated by Trizol reagent (Invitrogen) and cDNA was synthesized according to the manufacturer’s recommendations (Applied Biological Materials, Richmond, BC, Canada). RT-qPCR was subjected using EvaGreen 2X qPCR MasterMix (Applied Biological Materials). Expression levels were normalized to the amount of the β-ACTIN housekeeping gene. The following primers were used: IL-8 (forward, 5’-TGCACTTACTCTTGCCAGAACTG-3’, and reverse, 5’-CAAACTGGCTGTTGCCTTCTT-3’); IKKβ (forward, 5’-ACTTGGCGCCCAATGACCT-3’, and reverse, 5’-CTCTGTTCTCCTTGCTGCA-3’); β-ACTIN (forward, 5’-ATTGCCGACAGGATGCAGAA-3’, and reverse, 5’-GCTGATCCACATCTGCTGGAA-3’).

### Statistical Analysis

Each experiment was performed at least three times and data are presented as mean ± standard error of the mean (SEM). Statistical analysis was performed with IBM SPSS statistics 22. Differences between two groups were evaluated by independent Student’s t-test. *p* values less than 0.05 were considered statistically significant and are presented as *P < 0.05, **P < 0.01, ***P < 0.001 and ns, not significant.

## Results

### *spv* Inhibits NF-κB Activation During *Salmonella* Infection

**Figure 1:**
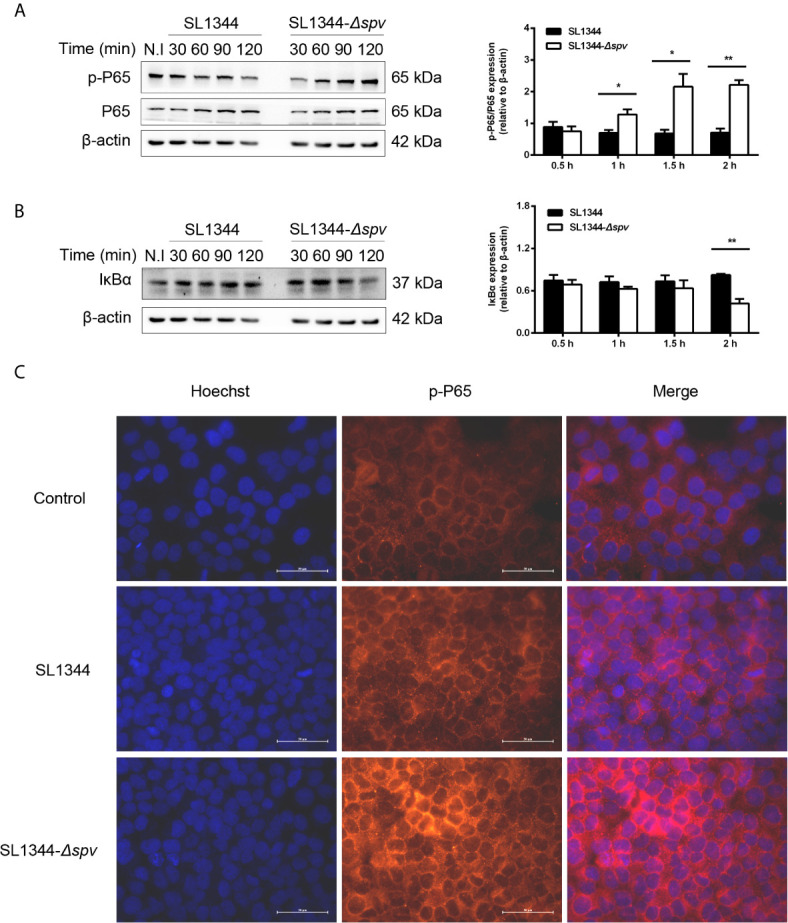
*spv* inhibits NF-κB activation during *Salmonella* infection. HeLa cells were infected with the WT or *Δspv S*. Typhimurium strain at an MOI of 100. **(A, B)** The cell lysates were prepared at the indicated time points and subjected to immunoblotting with anti-p-P65, anti-P65 **(A)** and anti-IκBα **(B)**. β-ACTIN was used as a loading control. Densitometric analysis of p-P65, P65 and IκBα relative to β-ACTIN protein and one of three representative Western blot experiments are shown. **(C)** Immunofluorescence microscopy of subcellular distribution of p-P65 in HeLa cells were visualized at 2 h post-infection. Original magnification, ×40; scale bars, 50 μm. One of three representative immunofluorescence microscopy experiments is shown. Statistical analysis was performed with IBM SPSS Statistics 22. Data were compared by independent Student’s t test. Values are expressed as mean ± SEM of three independent experiments. *p* values less than 0.05 were considered statistically significant and are presented as *P < 0.05 and **P < 0.01.

First, we investigated whether the *Salmonella spv* locus contributes to the intracellular *Salmonella* pathogenesis *via* interfering with the NF-κB pathway. Cells were infected with either the wild-type (WT) *S.* Typhimurium or the *Δspv* mutant *S*. Typhimurium strain to study the kinetics of p65 phosphorylation and IKBα degradation. We showed that cells infected with the *Δspv* strain displayed a significantly higher level of p65 phosphorylation compared with cells infected with the WT strain since 1 h post-infection (**Figure 1A**). Consistently, lower expression of IkBα was found in *Δspv*-infected cells compared with WT-infected cells at 2 h post-infection (**Figure 1B**). To confirm these results, immunofluorescence was used to assess the subcellular distribution of p-P65. The results showed that *spv* decreases the accumulation of p65 in the nucleus (**Figure 1C**). These findings indicated that *spv* inhibited the activation of NF-κB during *Salmonella* infection.

### SpvB Is Critical for *spv*-Mediated NF-κB Inhibition and IκBα Degradation

**Figure 2:**
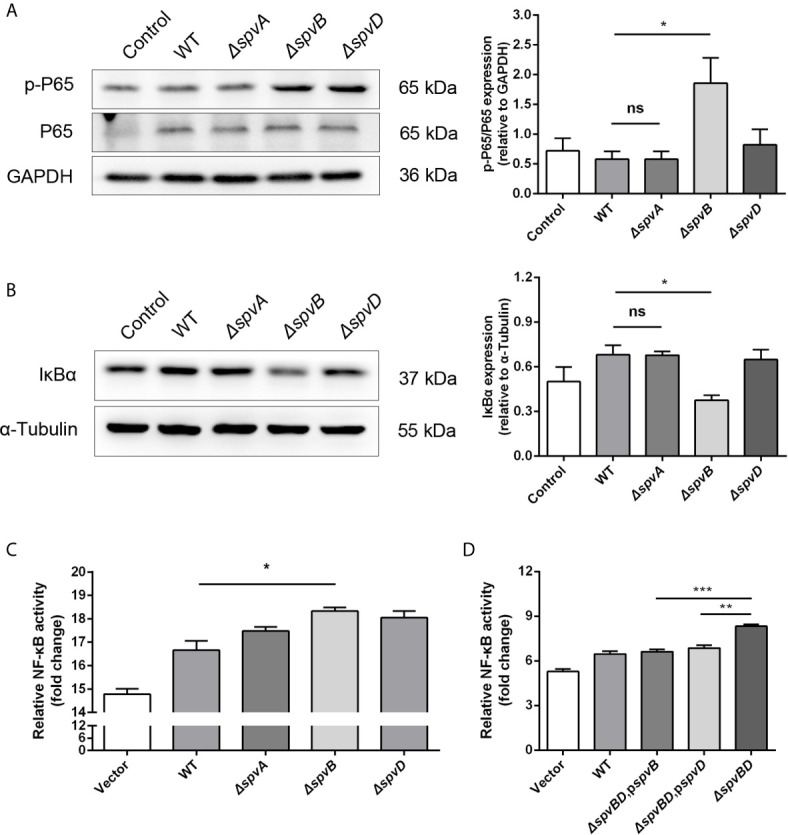
SpvB is critical for *spv*-mediated NF-κB inhibition and IκBα degradation. **(A, B)** HeLa cells were infected with the WT, *ΔspvA*, *ΔspvB* or *ΔspvD S*. Typhimurium strain at an MOI of 100 for 2 h. Whole cell lysates were analyzed by Western blot with anti-p-P65, anti-P65 **(A)**, anti-IκBα **(B)**, and anti-β-ACTIN antibodies (as loading control). Densitometric analysis of p-P65, P65 and IκBα relative to β-ACTIN protein and one of three representative Western blot experiments are shown. **(C, D)** Luciferase reporter assays of 293T cells transiently transfected with pNFκB-Luc (Firefly luciferase, experimental reporter) and phRL-TK reporter (Renilla luciferase, internal control). After 24 h, cells were infected with the WT, *ΔspvA*, *ΔspvB* or *ΔspvD S*. Typhimurium strain **(C)** and the WT, *ΔspvBD*, p*spvB*, *ΔspvBD*, p*spvD* or *ΔspvBD S*. Typhimurium strain **(D)** at an MOI of 100 for 2 h. All data were acquired at the same time. Statistical analysis was performed with IBM SPSS Statistics 22. Data were compared by independent Student’s t test. Values are expressed as mean ± SEM of three independent experiments. *p* values less than 0.05 were considered statistically significant and are presented as *P < 0.05, **P < 0.01, ***P < 0.001 and ns, not significant.

Our data showed that the whole *spv* locus contributed to IkBα degradation. However, previous studies have identified that SpvC does not affect NF-κB pathway, while SpvD prevents NF-κB activation by interfering with nuclear translocation of p65 but not degradation of IκBα (Rolhion et al., 2016). Therefore, we further studied the potential role of *spvA* and *spvB* in this process. We constructed *ΔspvA* and *ΔspvB* mutant *Salmonella* strains and assessed their roles in disturbing NF-κB activity, with *ΔspvD* mutant *Salmonella* strain as a control. There was no change in p65 phosphorylation level or IkBα expression in cells infected with the *ΔspvA* strain compared with cells infected with the WT strain at 2 h post-infection. Interestingly, the protein level of p-P65 in *ΔspvB*-infected cells was significantly higher than that detected in WT-infected cells, and the level of IkBα was markedly lower in cells infected with the *ΔspvB* strain (**Figures 2A, B**). Additionally, HeLa cells were transfected with plasmid expressing luciferase under the transcriptional control of NF-κB, and then infected with different *Salmonella* strains. NF-κB activity was measured at 2 h post-infection by luciferase reporter assay. The results showed that *ΔspvB*-infected cells exhibited an increased NF-kB activity, which is consistent with *ΔspvD*-infected cells, while there was no apparent difference in luciferase activity among *ΔspvA*- and WT-infected cells (**Figure 2C**). These observations suggested an important effect of SpvB on *spv*-mediated NF-κB inhibition. We further constructed *ΔspvBD* mutant *Salmonella* strain and the complemented *Salmonella* strains harboring *spvB* (*ΔspvBD*,p*spvB*) or *spvD* (*ΔspvBD*,p*spvD*). The data of luciferase assay showed that cells infected with *ΔspvBD*,p*spvB* or *ΔspvBD*,p*spvD* strain significantly reduced the NF-kB activity compared with those infected with *ΔspvBD* strain (**Figure 2D**). In line with the previous study (Rolhion et al., 2016), SpvD inhibited NF-kB activation (**Figure 2D**) but did not interfere with IkBα degradation (**Figure 2B**). Together, these results demonstrated that SpvB is critical for *spv*-mediated NF-κB inhibition and IκBα degradation.

### SpvB Inhibits IL-1β/LPS/TNF-α/PMA-Triggered Activation of the NF-κB Pathway

**Figure 3:**
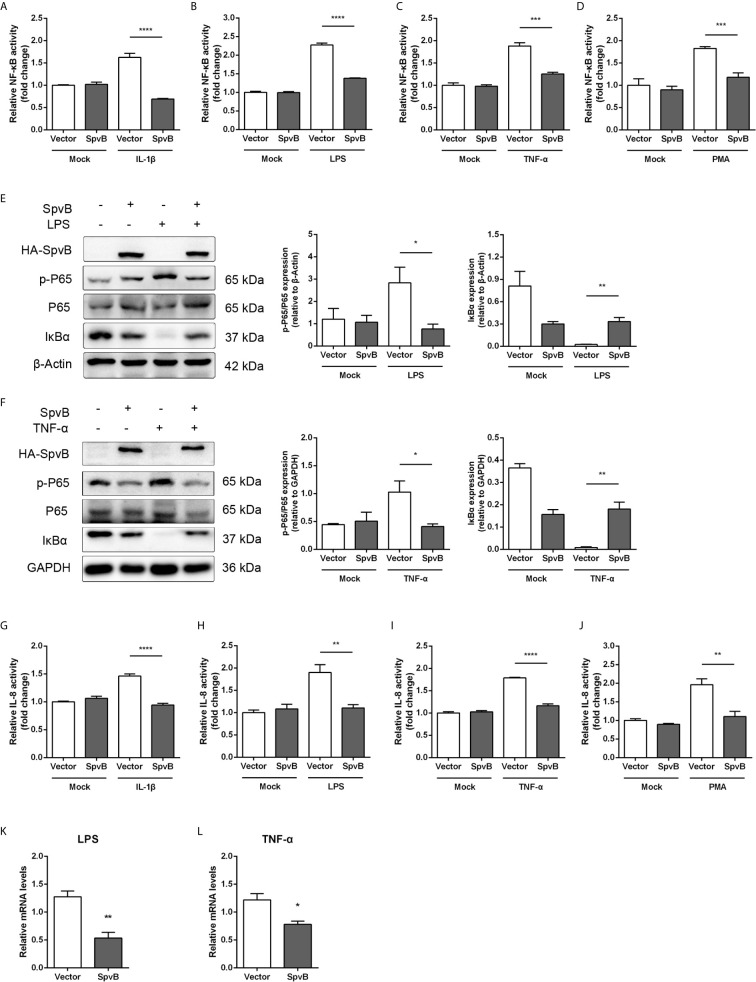
SpvB inhibits IL-1β/LPS/TNF-α/PMA-triggered activation of the NF-κB pathway. **(A–D)** Luciferase reporter assays of 293T cells transiently transfected with pNFκB-Luc, phRL-TK and a plasmid encoding SpvB-EGFP or the empty vector. After 24 h, cells were treated with IL-1β **(A)**, LPS **(B)**, TNF-α **(C)** or PMA **(D)** for 4 h and luciferase activity was measured. **(E, F)** HeLa cells transfected with HA-SpvB expressing plasmid or the empty vector were treated with LPS **(E)** or TNF-α **(F)** for 30 min prior to processing for Western Blot analysis of cell lysates with anti-p-P65, anti-P65 and anti-IκBα. **(G–J)** Luciferase reporter assays of 293T cells transiently transfected with pIL-8-Luc, phRL-TK and SpvB-EGFP expressing plasmids or the empty vector. After 24 h, cells were treated with IL-1β **(G)**, LPS **(H)**, TNF-α **(I)** or PMA **(J)** for 4 h, and luciferase activity was measured. **(K, L)** HeLa cells were transiently transfected with SpvB-EGFP expressing plasmid or the empty vector and stimulated with LPS **(K)** or TNF-α **(L)** for 4 h. IL-8 levels were determined by quantitative PCR. Values were normalized to those of the housekeeping gene β-ACTIN, and fold-changes relative to the untreated control are shown. Statistical analysis was performed with IBM SPSS Statistics 22. Data were compared by independent Student’s t test. Values are expressed as mean ± SEM of three independent experiments. *p* values less than 0.05 were considered statistically significant and are presented as *P < 0.05, **P < 0.01, ***P < 0.001 and ****p < 0.0001.

The NF-κB signaling pathway can be triggered by several cellular receptors during *Salmonella* infection, including IL-1R, TLRs, TNFR, and NLRs. To elucidate the underlying mechanisms by which SpvB inhibits NF-κB activity, the *spvB* gene was cloned into a mammalian expression vector pEGFP-N1 to generate HA-SpvB-EGFP fusion protein. After stimulation with IL-1β, LPS, TNF-α, or PMA, the NF-κB activity was measured in 293T cells with ectopic expression of SpvB using NF-κB-luciferase reporter assays. As shown in **Figures 3A–D**, the expression of SpvB efficiently blocked all four stimulants-induced NF-κB activation. In agreement with this observation, western blot analysis illustrated that the expression of HA-SpvB-EGFP, unlike the empty vector, decreased p65 phosphorylation and increased IkBα expression in the context of either LPS or TNF-α treatment (**Figures 3E, F**). Given that interleukin 8 (IL-8) transcription is regulated by NF-kB, we further analyzed the activity of IL-8 in 293T cells stimulated with IL-1β, LPS, TNF-α, or PMA using an IL-8 reporter assay to confirm SpvB-mediated inhibition of NF-kB activity. The results showed that IL-8 reporter activity in response to each of the four stimulants in cells transfected with SpvB-EGFP was significantly attenuated compared with mock transfected cells either stimulated with LPS or TNF-α (**Figures 3G–J**). Moreover, the mRNA level of IL-8 in cells expressing SpvB-EGFP was obviously lower than that detected in cells expressing EGFP (**Figures 3K, L**). Taken together, these findings indicated that SpvB inhibits the activation of NF-κB pathway triggered by all four stimulants.

### SpvB Prevents the Activation of NF-kB by Acting at IKKβ

**Figure 4:**
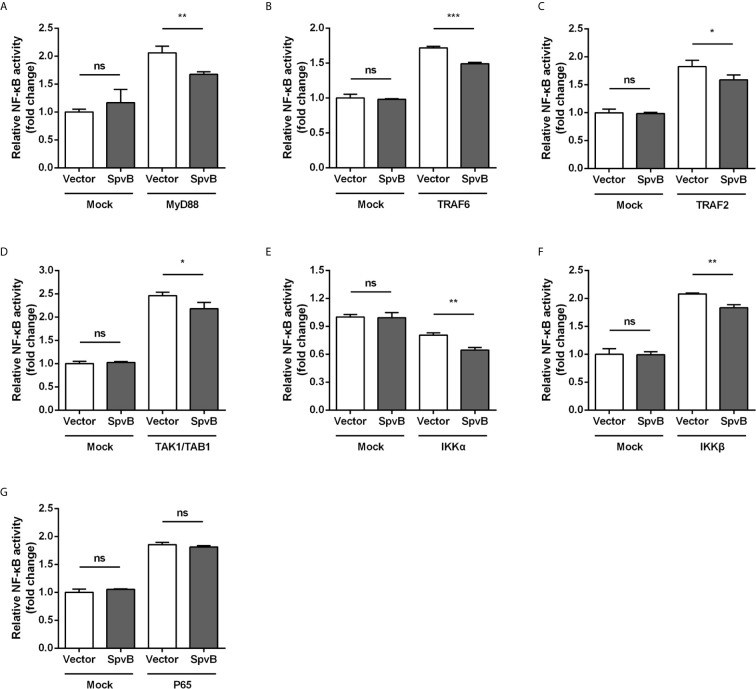
SpvB inhibits NF-kB stimulation upstream of P65. 293T cells were transiently transfected with an NF-κB reporter construct along with plasmids encoding MyD88 **(A)**, TRAF6 **(B)**, TRAF2 **(C)**, TAK1/TAB1 **(D)**, IKKα **(E)**, IKKβ **(F)**, or P65 **(G)** in conjunction with a plasmid encoding SpvB or the empty vector. The reporter activity was subsequently measured 24 h after transfection. Statistical analysis was performed with IBM SPSS Statistics 22. Data were compared by independent Student’s t test. Values are expressed as mean ± SEM of three independent experiments. *p* values less than 0.05 were considered statistically significant and are presented as *P < 0.05, **P < 0.01, ***P < 0.001 and ns, not significant.

To better understand the SpvB inhibitory mechanism and where in the NF-κB signaling pathway SpvB might exert its function, we tested whether SpvB itself was able to repress NF-κB activation in a context different from *Salmonella* infection. We also examined the effect of SpvB on the activation of NF-κB by several essential components of the signaling pathway. Thus, 293T cells expressing NF-κB luciferase reporter gene together with MyD88, TRAF6, TRAF2, TAK1/TAB1, IKKα, IKKβ, or P65 were transfected with SpvB-EGFP or the empty vector. As shown in **Figures 4A–F**, SpvB prevented the activation of NF-κB induced by expression of MyD88, TRAF6, TRAF2, TAK1/TAB1, IKKα, or IKKβ. In contrast, SpvB did not block the activation of NF-κB caused by expression of P65 subunit (**Figure 4G**). These results suggested that SpvB might exert its function at the level of IKKβ.

**Figure 5:**
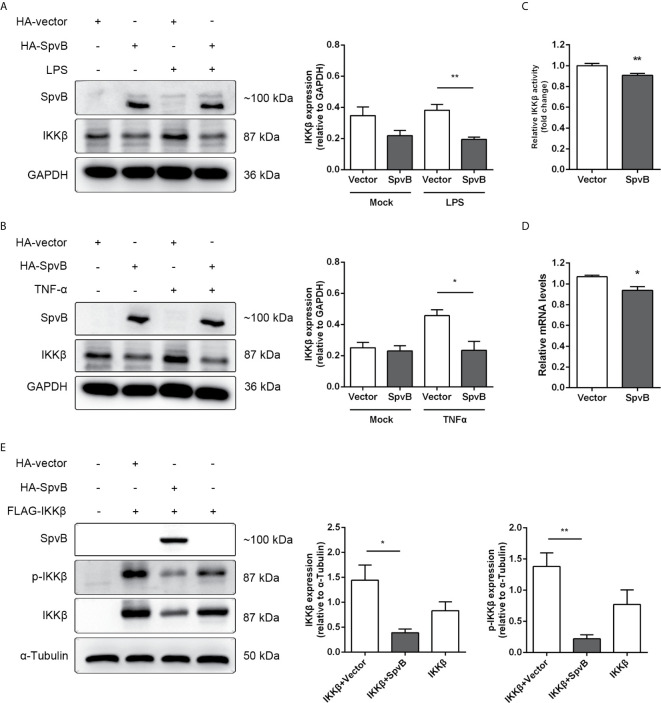
SpvB downregulates IKKβ expression and phosphorylation. **(A, B)** HeLa cells were transiently transfected with a plasmid encoding HA-tagged SpvB or the empty vector and stimulated with LPS **(A)** or TNF-α **(B)** for 4 h. Cell lysates were analyzed by Western blot with anti-HA, anti-IKKβ, and anti-GAPDH antibodies. Densitometric analysis of HA and IKKβ relative to GAPDH protein and one of three representative Western blot experiments are shown. **(C)** Luciferase reporter assays of 293T cells transiently transfected with pIKKβ-Luc, phRL-TK and SpvB-EGFP expressing plasmids or the empty vector. **(D)** HeLa cells were transiently transfected with SpvB-EGFP expressing plasmid or the empty vector. IKKβ mRNA level was determined by quantitative PCR. Values were normalized to those of the housekeeping gene β-ACTIN, and fold-changes relative to the untreated control are shown. **(E)** 293T cells were transiently transfected with FLAG-tagged IKKβ, and HA-tagged SpvB or the empty vector. Cell lysates were analyzed by Western blot with anti-HA, anti-pIKKβ, anti-IKKβ, and anti-α-Tubulin antibodies. Statistical analysis was performed with IBM SPSS Statistics 22. Data were compared by independent Student’s t test. Values are expressed as mean ± SEM of three independent experiments. *p* values less than 0.05 were considered statistically significant and are presented as *P < 0.05 and **P < 0.01.

We further studied the relationship between SpvB and IKKβ reduction. Western blot analysis showed that SpvB efficiently reduced the expression of IKKβ in context of either LPS or TNF-α activation (**Figures 5A, B**). Luciferase activity assay and quantitative PCR also confirmed that, even without stimulation, SpvB suppressed the promoter activity and mRNA level of IKKβ (**Figures 5C, D**). We further investigated the effect of SpvB on IKKβ by transiently co-expressing differentially epitope-tagged SpvB and IKKβ. The results showed that expression of SpvB led to a drastic reduction in the level of either IKKβ expression or IKKβ phosphorylation (**Figure 5E**). All these results indicated that SpvB downregulates NF-kB activity *via* acting on IKKβ.

### SpvB Inhibits IKKβ Expression and Phosphorylation by Targeting KEAP1

**Figure 6:**
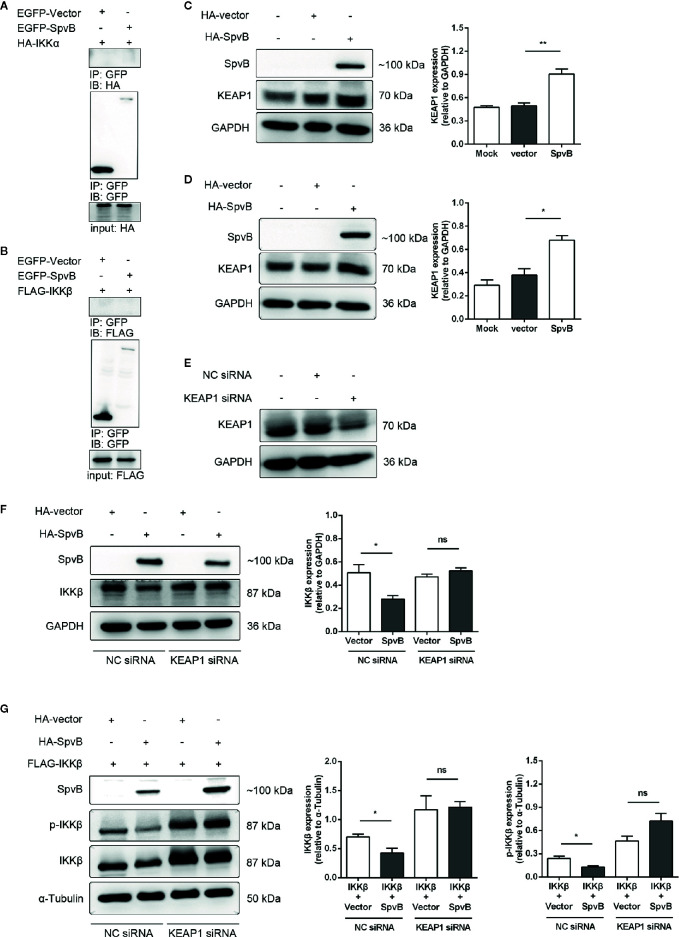
SpvB inhibits the expression and phosphorylation of IKKβ by targeting KEAP1. **(A, B)** 293T cells were transfected with HA-tagged IKKα **(A)** or FLAG-tagged IKKβ **(B)** with or without SpvB-EGFP. After 24 h, cells were harvested and subjected to co-immunoprecipitation. HeLa cells **(C)** and 293T cells **(D)** were transiently transfected with HA-tagged SpvB or the empty vector. Whole cell lysates were analyzed by Western blot with anti-HA, anti-KEAP1, and anti-GAPDH antibodies. **(E)** 293T cells were treated with KEAP1 siRNA or the negative control for 48 h prior to processing for Western Blot analysis of cell lysates with anti-KEAP1 and anti-GAPDH antibodies. **(F)** HeLa cells were transiently transfected with HA-tagged SpvB or the empty vector. After 24 h, cells were treated with KEAP1 siRNA or the negative control for another 24 h. Whole cell lysates were analyzed by Western blot with anti-HA, anti-IKKβ, and anti-GAPDH antibodies. **(G)** 293T cells were transiently transfected with FLAG-tagged IKKβ and HA-tagged SpvB or the empty vector. After 24 h, cells were treated with KEAP1 siRNA or the negative control for another 24 h. Cell lysates were analyzed by Western blot with anti-HA, anti-pIKKβ, anti-IKKβ, and anti-α-Tubulin antibodies. Statistical analysis was performed with IBM SPSS Statistics 22. Data were compared by independent Student’s t test. Values are expressed as mean ± SEM of three independent experiments. *p* values less than 0.05 were considered statistically significant and are presented as *P < 0.05, **P < 0.01 and ns, not significant.

We next focused on elucidating the mechanisms underlying SpvB-mediated downregulation of IKKβ. We first investigated whether SpvB directly interacted with IKKα or IKKβ. To this end, we transfected EGFP-tagged SpvB together with HA-tagged IKKα or Flag-tagged IKKβ into 293T cells to analyze molecular interactions by co-immunoprecipitation (co-IP). The results exhibited that neither IKKα nor IKKβ could bind to SpvB (**Figures 6A, B**). In previous studies, we found that SpvB interferes with intracellular iron homeostasis *via* regulation of a transcription factor Nrf2 (Yang et al., 2019). It is known that the cytoplasmic protein KEAP1 interacts with Nrf2 and represses its function, which suggests that SpvB might positively modulate KEAP1 to downregulate IKKβ and suppress NF-κB activity. To address this issue, 293T cells or HeLa cells were transfected with HA-tagged SpvB or the empty vector, and western blot was employed to analyze the effect of SpvB expression on KEAP1. Data showed that the expression of SpvB significantly increased the level of KEAP1 (**Figures 6C, D**). Therefore, we speculated that SpvB might inhibit the expression and phosphorylation of IKKβ by affecting KEAP1. To further characterize the effect of KEAP1 in this process, 293T cells or HeLa cells were transfected with HA-tagged SpvB or the empty vector, and then treated with KEAP1 siRNA or the negative control (NC siRNA). Western blot analysis illustrated that SpvB potently reduced the expression of IKKβ following the treatment of NC siRNA, whereas this reduction was dissolved in the context of KEAP1 siRNA treatment (**Figures 6E, F**). To determine the phosphorylation of IKKβ, FLAG-tagged IKKβ and HA-tagged SpvB or the empty vector were co-transfected into 293T cells, and the cells were treated with KEAP1 siRNA or NC siRNA. Western blot analysis showed that in NC siRNA treatment group, SpvB not only decreased the expression of IKKβ but also restrained the phosphorylation of IKKβ. Conversely, whether SpvB was expressed or not, there was no significant difference in the expression or phosphorylation of IKKβ following the treatment by KEAP1 siRNA (**Figure 6G**). All these observations indicated that SpvB inhibits IKKβ expression and phosphorylation by targeting KEAP1.

## Discussion

Bacterial pathogens have a broad arsenal of genes that are tightly regulated and coordinated to facilitate adaptation to the host environment (Ilyas et al., 2017). *S*. Typhimurium, a facultative intracellular Gram-negative pathogen causing both gastrointestinal and systemic diseases in humans and other mammals, primarily depends on two T3SSs encoded within SPI-1 and SPI-2 to deliver effectors into host cells to alter cell physiology for bacterial invasion and colonization (Jennings et al., 2017; Lou et al., 2019). Emerging evidence indicates that *Salmonella* effector proteins are utilized by bacteria to modulate host inflammatory response and prolong intracellular bacterial survival. A few effectors have been shown to have an impact on the activity of key signaling pathways such as NF-κB, signal transducer and activator of transcription 3 (STAT3), and MAPK, which result in reduced subsequent production of pro-inflammatory cytokines and/or suppressed host inflammation. For example, SseK1 and SseK3 inhibit NF-κB signaling and necroptotic cell death in *Salmonella*-infected macrophages (Gunster et al., 2017), while PipA, GtgA, and GogA are proteases that target NF-κB transcription factors to preserve host homeostasis (Sun et al., 2016). AvrA inhibits MAPK pathway in epithelial cells *via* a JNK-dependent manner (Collier-Hyams et al., 2002; Jones et al., 2008), whereas SteE drives M2 macrophage polarization *via* GSK3 and STAT3 (Panagi et al., 2020). Nonetheless, the function of most *Salmonella* effectors is still unclear.

Previous studies have reported that *S*. Typhimurium carries *spv* genes, highly conserved in the pSLT virulence plasmid and closely related to bacterial pathogenicity, have been implicated in suppressing host innate immune response against bacterial infection (Wu et al., 2016). However, the mechanisms through which *spv* affects host innate immune response remain elusive. Since NF-κB signaling pathway is one of the central host defense responses to limit *Salmonella* infection, we hypothesized that *spv* may restrain host inflammation and promote *Salmonella* infection by efficiently attenuating NF-κB activation. Consistent with this hypothesis, we found that *spv* contributed to the inhibition of the phosphorylation of p65 and the degradation of IκBα, indicating that Spv could negatively regulate NF-κB signaling during *Salmonella* infection. The *spv* locus contains five genes, designated as *spvRABCD*. The SpvB, SpvC, and SpvD proteins are thought to be delivered into the host cells mainly through the SPI-2 T3SS and have been proven as virulence effectors in a variety of cell types. It has been reported that SpvC has phosphothreonine lyase activity on host MAPK but no effect on NF-kB signaling pathway (Mazurkiewicz et al., 2008). SpvD was found to inhibit NF-κB activation by prevention of nuclear transport of p65 but not degradation of IκBα (Rolhion et al., 2016). In this study, we found that *spv* inhibited IκBα degradation, which suggests that *spv*-mediated NF-kB inhibition might depend on SpvA or SpvB instead of SpvC or SpvD. Therefore, we generated deletion mutants for *spvA* or *spvB* to identify their effect on the activation of NF-kB and constructed *spvD* mutant strain as a control.

Our data showed that *spvB*, rather than *spvA*, was critical for the inhibition of NF-kB activation. Furthermore, a double mutant strain lacking both *spvB* and *spvD* exhibited significantly stronger activation of the NF-κB reporter than each of the single mutants complemented with either *spvB* or *spvD*, suggesting that these effectors are not functionally redundant. SpvB has been characterized as an ADP-ribosylase, which can prevent actin polymerization, thereby leading to loss of the actin cytoskeleton (Guiney and Fierer, 2011). SpvD is a cysteine hydrolase with a serovar-specific polymorphism (Grabe et al., 2016). Both effectors are likely to have distinct biochemical activities and physiological effects during *Salmonella* infection. Nathalie R et al. have demonstrated that SpvD disrupts normal recycling of importin-α from the nucleus, leading to a defect in nuclear translocation of p65 and inhibition of activation of NF-κB-regulated promoters, while it does not lead to the degradation of IκBα (Rolhion et al., 2016). In this context, we further investigated the mechanism by which SpvB inhibits the IκBα degradation and NF-κB activation.

NF-κB is a transcription factor that is sequestered in the cytosol by IκBα in resting cells, and multiple exogenous and endogenous stimuli are needed to induce its translocation to the nucleus. In canonical NF-κB signaling pathway, cellular receptors including TNFR, IL-1R, TLRs, and NLRs sense different stimuli, and then utilize various adaptors and signaling molecules to transmit signals to initiate the phosphorylation of IKKs, degradation of IκBα, nuclear transfer of p65, and final activation of NF-κB signaling pathway (Bhoj and Chen, 2009). To characterize the SpvB-dependent dampening of NF-kB activation, we employed NF-kB-luciferase reporter assays to measure the levels of NF-kB activity in SpvB-expressing 293T cells that were stimulated with TNF-α, IL-1β, LPS, or PMA. Interestingly, we found that SpvB blocked NF-κB activation induced by all these stimuli. Consistent with this, SpvB restrained the activated IL-8 expression without specificity. Given that the inhibition of NF-κB signaling mediated by the ectopic expression of SpvB is independent of different stimuli, we speculated that SpvB might act on the components of their common downstream pathway (Ruchaud-Sparagano et al., 2011).

We subsequently investigated which of the components function as a potential SpvB target. Previous studies have shown that MyD88 and TRAF6 were downstream of the IL-1β receptor, and TRAF2 was the TNF-α receptor. Here we found that SpvB inhibition of NF-κB reporter activity was driven by expression of MyD88, TRAF6, and TRAF2. Importantly, we observed that SpvB downregulated NF-κB activity stimulated by TAK1/TAB1, IKKα, or IKKβ downstream of TRAF, but not by P65 expression. These findings suggested that SpvB might affect IKKβ activity. The following results showed that SpvB not only inhibited the transcription and expression of IKKβ but also suppressed the phosphorylation of IKKβ. All these results indicated that SpvB directly or indirectly targets IKKβ to exert the inhibitory effect on NF-kB activity. To elucidate the underlying modulatory mechanism, we further performed immunoprecipitation assays to determine whether IKK complex can be physically associated with SpvB. However, SpvB failed to bind with either IKKα or IKKβ.

Our previous study reported that SpvB facilitates the pathogenicity of *Salmonella* within cells *via* disrupting host iron metabolism by targeting the transcription factor Nrf2 (Yang et al., 2019). In mammals, Nrf2 interacts with the E3 ligase adaptor KEAP1, the major endogenous negative regulator of Nrf2, to form a defense system aimed to preserve cellular homeostasis (Bellezza et al., 2018). In fact, KEAP1 also functions as an E3 ubiquitin ligase to dysregulate IKKβ degradation and phosphorylation (Lee et al., 2009). In view of the Nrf2 degradation enhanced by SpvB, we detected the expression of KEAP1 in cells transiently transfected with HA-tagged SpvB, and our data showed that SpvB significantly increased the expression of KEAP1. We further investigated whether the *Salmonella* effector SpvB inhibits the expression of IKKβ by modulation of KEAP1. Cells expressing SpvB were treated with KEAP1 siRNA or NC siRNA. Western blot analysis illustrated that SpvB reduced the expression of IKKβ following the treatment with NC siRNA. However, there was no significant difference in IKKβ level between SpvB- and vector-transfected cells following KEAP1 siRNA treatment. All these findings indicated that the *Salmonella* effector protein SpvB can prevent IKKβ expression and NF-kB activation by targeting KEAP1. Moreover, our data confirmed that SpvB restrains the phosphorylation of IKKβ through KEAP1. Wei et al. recently reported that S53 is a potential phosphorylation site of KEAP1 and its phosphorylation is critical to the activity of KEAP1 (Wei et al., 2019). It is possible that SpvB upregulates the expression of cytosolic KEAP1, and the latter competes with IKKβ for phosphate groups in order to maintain its own activity, which leads to the inhibition of NF-kB signaling pathway.

Together, our work revealed a novel mechanism for a *Salmonella* effector to inhibit NF-kB activity as it was demonstrated that *Salmonella* effector SpvB has a potent and specific ability to prevent activation of NF-κB *via* suppressing IKKβ activity by targeting KEAP1. Even though inflammation is ultimately induced by *Salmonella* infection, SpvB may allow *Salmonella* to delay host immune response to establish infection and disseminate to other tissues. These findings revealed a remarkable adaptation of a bacterial pathogen to promote its own survival and virulence.

## Data Availability Statement

The raw data supporting the conclusions of this article will be made available by the authors, without undue reservation.

## Author Contributions

SY, QD, and YL designed and conducted the experiments. SY, QD, and YL contributed to development of methodology. SY, QD, LS, YZ, KD, and YL contributed to analysis and interpretation of data. SY, QD, and LS prepared figures and wrote the manuscript. SW, RH, and YL supervised the project and edited the manuscript. All authors contributed to the article and approved the submitted version.

## Funding

This work was supported by National Natural Science Foundation of China (no. 31970132, no. 81971899), Suzhou Municipal Science and Technology Foundation (SYS2019031), and a project funded by the Priority Academic Program Development (PAPD) of Jiangsu Higher Education Institutions.

## Conflict of Interest

The authors declare that the research was conducted in the absence of any commercial or financial relationships that could be construed as a potential conflict of interest.

## Abbreviations

co-IP , co-immunoprecipitation; DMEM, Dulbecco’s modified Eagle’s medium; FBS, fetal bovine serum; IL-8, interleukin 8; IL-1R, interleukin-1 receptor; IKKβ, IκB kinase β; LB, Luria-Bertani; MAPK, mitogen-activated protein kinases; MOI, multiplicity of infection; NC siRNA, negative control siRNA; IκBα, NF-κB inhibitor alpha; NLRs, NOD-like receptors; NF-κB, nuclear factor κB; IKKs, nuclear factor-κB kinases; SPI-1, pathogenicity islands-1; SPI-2, pathogenicity islands-2; RT-qPCR, Quantitative real-time PCR; *S*. Typhimurium, *Salmonella enterica* serovar Typhimurium; *spv*, *Salmonella* plasmid virulence; STAT3, signal transducer and activator of transcription 3; SEM, standard error of the mean; TLRs, toll-like receptors; TNFR, tumor necrosis factor receptor; T3SS, type III secretion system; WT, wild-type.
